# Dr. Cheyne on Malingering

**Published:** 1828-04-01

**Authors:** 


### *On Malingering; or, feigned Diseases.* By Dr. CHEYNE.

[Dublin Hospital Reports.]

MAN'S journey through life is a perpetual contest between pleasure and pain. He is constantly grasping at the former, and endeavouring to ward off the latter. In the eager pursuit of pleasure, he will frequently undergo a very long course and intense degree of pain—though the former is often transient, or even nugatory, when obtained; while the latter is always real, and not seldom destructive, before the object for which it is encountered can be accomplished. It is difficult to say whether war or peace be the condition designed for mankind by Nature. If we consult history, we shall find that the former has predominated over the latter—and if we examine minutely into the constitution of the human mind, and the play of human passions, we shall have little ground for anticipating the promised milennium, under the present dispensation of things! But leaving speculations of this kind to the NATURAL philosopher, who is often imperfectly acquainted with HUMAN NATURE, we may remark that the medical philosopher, whose destinies lead him into naval and military life, comes in contact with no small number of sailors and soldiers who, in battle, will excel the heroic actions of Achilles and Hector; but, when the day of carnage is past, will undergo tortures inconceivable, to get out of the "paths of glory," in order to fall back into the dull and insipid routine of civil life. It is nearly two thousand years since Horace humorously described the FACT of "nemo contentus," but he has not been very happy in ascertaining the true cause. Were we disposed to enter into metaphysical, or rather physical investigations, we think we could elucidate the etiology of this contrariety in human nature, by somewhat more satisfactory reasons than the Roman satirist has adduced; but into these domains we dare not venture in this place.

We do not think it worth while to search the records of antiquity—or even Dr. Good's "Study of Medicine," to ascertain whether a Chaldaic, Arabic, Greek, or Roman name, has been given to a class of diseases which have more precise and determinate causes than any in the whole range of nosology. Neither is the disease rare or uncommon. It has prevailed in all ages of the world, and among all classes of society. What was the long INDISPOSITION of ACHILLES, at the seige of Troy? A huge fit of MALINGERING! But Achilles was a great man, and his malingering nearly ruined the Greek cause—therefore he has been rewarded with immortality in the deathless verse of Homer! Malingering indeed is not confined to the RANKS in the army—nor is it only to be found BEFORE THE MAST in our fleets. When an officer finds a ship or a regiment uncomfortable, he is seized with a fit of malingering—and is removed to another. When the statesman finds the helm of politics unmanageable, he is attacked with the malingering malady—or his wife is attacked with it, which is the same thing, and he quits his post. Nay, there is scarcely a private family in which the medical practitioner will not occasionally meet with a sulky Achilles or a foreboding Cassandra, malingering for some secret purpose. In these last cases, however, there is little mischief done. If the doctor is deceived, and prescribes strong medicines, the patient takes good care to throw them into the fire—if he detects the imposture, he prescribes some very pleasant julep, pockets his fee, and the malingering fit is allowed to take its course. But it is very different in the public service. The naval or military surgeon must decide between real and assumed diseases—between those produced by inevitable causes, and those induced by art, for the purpose of deception. And truly this is no easy task. It is difficult to discriminate between an ulcer kept up by irritating substances clandestinely applied, and one that is maintained by a peculiar habit of body. The moral evidence of malingering is not to be got. There is a free-masonry among soldiers and sailors that completely seals their mouths against any thing like blabbing the secrets of their messmates. Indeed there is great reason to believe that, in some regiments, the methods of malingering have been regularly systematised, and handed down as heir-looms for the benefit of those who may be inclined to make trial of them.

The following picture will shock the eyes of our innocent civilians, who look up to the heroic soldier as the BEAU IDEAL of human valour and simplicity of heart.

"As allusion has been made to ophthalmia, I may take the present opportunity of observing, that I never saw a more humiliating picture of depravity, or perversion of reason, call it what we may, than I have witnessed in a ward filled with soldiers labouring under that disease; most of the cases, as I learnt from the surgeon in attendance, being factitious. The methods, by which inflammation of the eye is produced and maintained, have not all been brought to light, but quick lime, infusion of tobacco, the gonorrhœal discharge, cantharides ointment, nitrate of silver, blue stone, and other metallic salts, are probably among the most common irritants employed. Inflammation thus caused is most painful, and is kept up under every privation which can make life miserable: locked up in a dark ward, and permitted to have intercourse only with the officers of the hospital, nurses and orderlies, confined to diet which, from the absence of every stimulating material, is most disrelishing, suffering under painful external applications, and nauseating internal medicines, phlebotomized and leeched till their complexions are bloodless, their pulse hæmorrhagic, and the frightful train of nervous symptoms, which excessive bloodletting produces, is established in the system.—All these evils, in many cases, have no effect but to confirm the soldier in his determination to destroy one or both of his eyes, that he may be dismissed from the service with the chance of a small pension.

"Wonderful indeed is the obstinacy which some malingerers evince. Night and day they will remain with the endurance of a fakir, in a position the most irksome. For weeks or months many men have, with surprizing resolution, sat and walked with their body bent double. Some have continued to irritate sores in the leg until the case became so bad as to require amputation of the limb, and many instances have occurred, in Military and Naval hospitals, of factitious complaints ending fatally."

The medical officer is placed in an awkward predicament in many cases. Men have been suspected of malingering, when concealed disease was going on internally, and punishment has been inflicted where the unfortunate sufferer has been labouring under natural afflictions. Thus a soldier of the 9th regiment of foot, who complained of great uneasiness in the loins, was treated as a malingerer, "and was sent to punishment drill, at which he was kept till a tumour appeared in his back, symptomatic of a lumbar abscess, of which the poor fellow died." Humanity shudders at such a history! But we must now proceed to notice some specimens of malingering, which will not be devoid of physiological interest.

1. *Paralysis.* This is often feigned by the soldier. Dr. Cheyne augurs that it is so, when it comes (or is said to come) on suddenly in *one* member, the rest of the body being free—the health good—and the sensorial functions in a state of integrity. These reasons are not proof positive, in our minds, though they may fairly lead to suspicion in military life. Smart shocks of electricity generally cure these malingerers. Some, however, have had stoicism enough to resist all electric batteries. A trooper of the 12th pretended he had lost the use of his right arm, and, after numerous attempts to detect or cure him, he was discharged. When fairly seated on the top of the coach, he waved the paralytic arm in triumph, and cheered at the success of his plan.

"A militia soldier, who pretended that he had lost the use of his inferior extremities, was reported unfit for service, by the late Dr. Harvey, and discharged. When he had obtained possession of his discharge, he caused himself, on a field day, to be taken in a cart to the Phœnix Park, and in front of the regiment, which was drawn up in line, he had the cart driven under a tree, upon which he hung up his crutches, leaped out of the cart, sprung three times from the ground, turned his back to the regiment, and having slapt his breech, he scampered off at full speed."

Surely there must be a defect in the laws to allow such scandalous proclamation of persevering falsehood and deception.

2. *Deaf and Dumb Malingerers.* As those who are born deaf never acquire speech, however perfect may be the organs of the latter faculty, so the soldier, thinking that these two defects must be necessarily connected, pretends to lose the two faculties at the same time. This, in itself, Dr. Cheyne thinks a sufficient proof of imposture; as it is improbable, in the highest degree, that paralysis of the two sets of organs should occur at the same instant, the health and mental faculties being otherwise good.

3. *Eneuresis,* or incontinence of urine, is a common case of malingering. It is detected by giving a large dose of opium in disguise, when the malingerer will fall asleep, and pass the whole night without wetting his bed.

4. *Epilepsy.* This disease is often simulated, and is sometimes extremely difficult of detection. The author relates a case, where a soldier feigning a paroxysm, was unmasked by an assistant putting some spirituous tincture into the man's eyes. He could not bear the pain of this—suddenly left off his contortions, and jumped up, to the no small amusement of the bye-standers.

5. *Insanity* is a species of malingering which is equally difficult to feign and detect. Dr. Cheyne gives a long list of distinctions between real and counterfeited insanity, some of which are curious, if not whimsical. Thus, he says, there is often, in the nature of this paroxysm, something which is inimitable—as, for example, "an astonishing power of stringing rhymes together." This probably explains the near alliance of poetry to madness.

6. *Hæmoptoe* is rather a favourite disease with soldiers who wish to obtain their discharge. It is detected by careful examination of the chest, and of the attendant symptoms. The complaint is feigned by bringing blood, by means of small punctures, from some part of the mouth or nose, as from the gums, fauces, Schneiderian membrane, &c.

7. *Phthisis.* Who would expect that this disease should be selected for imitation! Yet the malingerer "will often undertake a perfect portrait of that disease, and this he will sometimes execute with great cleverness." These people shew themselves more attentive students of symptomatology than some of their medical attendants.

8. *Palpitation of the Heart.* This is produced by taking white hellebore, as proved by Mr. Copland Hutchison.

We shall conclude this article with the following extract, which may be useful to the routine practitioner, who pins his faith on the doctrines and practice of Abernethy, to the ruination of hundreds of stomachs annually.

"Perhaps I may be permitted to remark that the present treatment, adopted by many civil as well as military practitioners of medicine, for the cure of supposed liver complaints, or even of actual liver complaints of a slight kind, is sometimes productive of very sad consequences. Many a course of mercury is undergone, to the indescribable discomfort of the patient, for pains seated in the intestines, in the duodenum or colon, perhaps in the biliary ducts, or even in the liver itself, which would have yielded to cupping, blistering, common purgatives, and a change of regimen. Thus, for example, there is a pain in the right hypochondrium which belongs to hysteria, which will yield to aloetic purgatives, the belladonna plaister, to infusion of valerian and snake root with ammonia, change of residence, exercise in an open carriage or on horseback, and light animal food without wine, which has entailed on many a sufferer, not one, but repeated courses of mercury, each in succession tending more and more to confirm the pain, till at last, by these means, the comforts and prospects of the patient have been utterly destroyed. Moreover, a great proportion of the cases of dyspepsia, which are generally treated on what is called Mr. Abernethy's plan, may be removed, with equal certainty, without giving a grain of mercury, by means of a pill every second evening, containing aloes, if it agrees, if not, a pill which will act slowly and moderately, a draught before meals containing some bitter infusion with an alkali, or some nervous medicine, carminative, or chalybeate, according to the case, appropriate diet and regimen, and change of residence. My opportunity of observing most of the varieties of disordered digestion, and my experience in treating them without mercury, leads me to protest against the present routine of practice in these cases."

It would be a merciful dispensation of Providence, if every young practitioner were to have one year's misery of dyspepsia—say the first year, when he would have plenty of time to attend to himself! It would save some thousands of his future dyspeptic patients from the murderous practice of blue pill at night, and black-broth in the morning—a practice which we verily believe inflicts a greater annual amount of moral and physical sufferings on humanity, than all the other "errors of medicine" put together! We seriously advise the rising generation of the profession to look to this in time. The non-professional public is every day acquiring experience on this point; and we have the means of knowing, that an important revolution is on the eve of bursting forth, in respect to the propriety of deluging the dyspeptic stomach with drastic medicines.

